# Supplementary material for: History of episodic heavy alcohol use predicts antidepressant effectiveness of ketamine
Source: Psychopharmacology (Berl). 2025 Oct 8;243(7):1693–9. doi: 10.1007/s00213-025-06918-4 (PMC13284259; doi:10.1007/s00213-025-06918-4)
Supplement: Supplementary file 1 [file 213_2025_6918_MOESM1_ESM.pdf]

Response to reviewers

Dear Editors and Reviewers:

Thank you for a thoughtful and comprehensive review of our manuscript, "History of episodic heavy alcohol use predicts antidepressant effectiveness of ketamine." We appreciate the positive overall comments, and have made the reviewers' recommended changes below:

Reviewer #1:

This is a retrospective analysis of patients with trMDD who received treatment with IV ketamine or ECT at McLean Hospital, and explored depressive outcomes (QIDS) as a function of the magnitude of heavy alcohol use over the past year (assessed with the AUDIT). The attempt to identify clinical features which may support a precision approach for TRD therapeutics is important. In that light, this report significant adds to this literature and supports the notion that a past year history of heavy drinking is associated with a more favorable and specific response to IV ketamine, as no such relationship was observed in the much larger ECT cohort.

The manuscript is very well written. The analyses are sound. The discussion is comprehensive, and notes several important limitations (non-randomization, retrospective, potential selection biases, lack of characterization of AUD diagnosis, etc). There is an appropriate discussion of potential NMDA receptor-mediated mechanisms underlying this association.

Thank you for the overall positive comments about the analyses, impact, and writing quality. We have addressed specific comments below.

There are a few issues to address which will improve the manuscript:

1) Please specify if the QIDS-SR or QIDS-C was used throughout.

We have changed all instances of "QIDS" to "QIDS-SR" and defined this abbreviation the first time it is presented in the abstract and the main text.

2) Please report the average mg/kg dose across the total Ketamine group, and if possible, within the separate AUDIT groups. It appears that all patients were started at 0.5 mg/kg and could be titrated up to 1.1 mg/kg at the clinician's discretion. It would be of high interest to analyze whether the heavy drinkers (AUDIT groups 3-4), required higher doses of IV ketamine compared to the light or no drinking groups. Did this dose optimization and titration in heavy drinkers in fact explain the better response compared to low AUDIT

groups? Ofcourse with only 49 subjects with AUDIT scores greater than zero, this type of analysis is limited by sample size, but it is worth exploring.

This is a good idea, but unfortunately dosing was not systematically recorded in the database that was analyzed in the present study. We have added this to the limitations section (new text underlined):

The retrospective nature of this study comes with limitations. A direct comparison between ECT and ketamine was not conducted as we would be unable to control for selection bias. For instance, the patients may differ in terms of treatment resistance, severity, and socioeconomic status amongst other variables. Similarly, there may be other differences between patients with and without a history of alcohol use, which may have affected other treatment-related parameters – for instance, patients with a history of AUD may tolerate higher doses of ketamine. Ketamine is also more novel and more expensive, which may lead to greater nonspecific effects such as expectation bias. It should also be noted that we have distinguished between Episodic Heavy Alcohol Use and Alcohol Use Disorder; full diagnosis of AUD is difficult to assess because patients with comorbid AUD have been either systematically excluded or underrepresented in many prospective studies of ketamine (Murrough et al. 2013; Daly et al. 2018). This trend also applies the present sample, which excluded patients who met formal criteria for an active substance use disorder. Furthermore, ketamine may be less accessible to patients with AUD due to high out-of-pocket costs or required social and financial supports to attend treatments. Thus, patients with comorbid AUD are likely underrepresented in the current sample, suggesting that our reported effect is likely an underestimate. Future prospective studies, particularly those comparing ketamine to ECT, may further address this question by actively enrolling patients with and without comorbid AUD. Future studies may gain additional insights with sophisticated phenotyping, including remission status and duration, previous and concurrent recovery treatment activities, medication-assisted treatment, history of binge drinking versus daily drinking, length of time since last alcohol use, presence and nature of cravings, and other phenomenological aspects of AUD. More pronounced effects may also be detected with genotyping or assessment of synaptic plasticity with brain stimulation and/or NMDAR modulating drugs.

3) Table 1 is missing the row for "Race/ethnicity"

This has been corrected:

Race/ethnicity<br>*(multiple selections allowed)*	*89% White*<br>*1% Native American*<br>*3% Asian/Pacific Islander*<br>*2% Black*<br>*3% Latino*<br>*2% Other*	*92% White*<br>*1% Native American*<br>*6% Asian/Pacific Islander*<br>*0.5% Black*<br>*2% Latino*<br>*2% Other*	*n/a*

4) Figure 1: please provide the N's for each AUDIT-3 score group, as well as for the binarized classification.

We have added the N as requested:

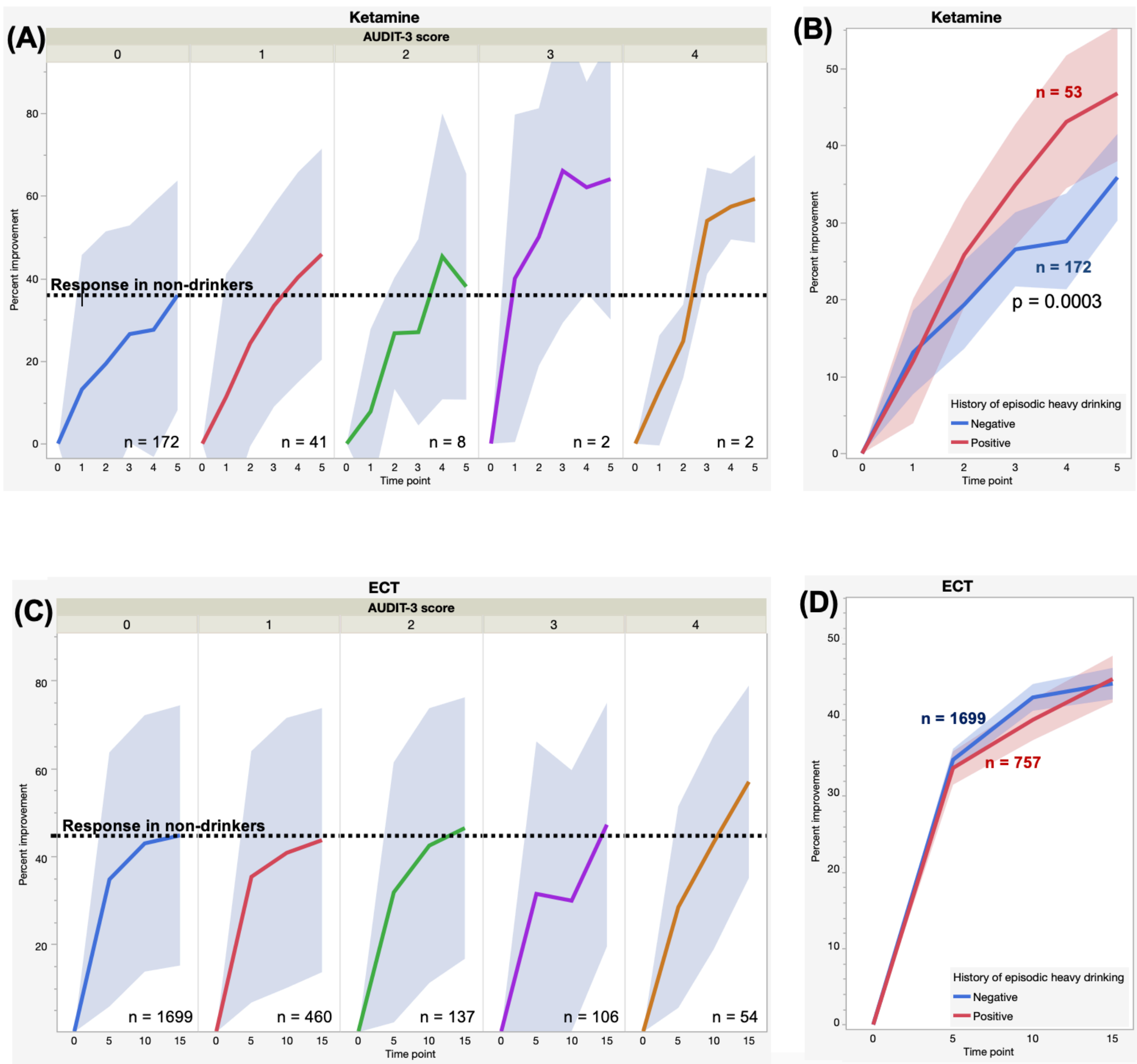

Reviewer #2: The authors investigated the effect of alcohol use history on treatment response to ketamine and ECT treatment in a retrospective cohort of open label real-world data. They found that patients who reported higher alcohol use over the past year

pretreatment showed better response ketamine. Similar patterns were not observed in the ECT cohort.

The manuscript is easy to read. The research question is of interest to the field. The analyses are reasonable. The results are informative. The real-world data and large sample are a strength. These findings are considered pilot and should be confirmed in future RCT. Overall, the study and its findings are a good addition to the literature.

Thank you for the positive overall comments. We have addressed the reviewer's specific recommendation below.

Table 1 should provide value per alcohol use subgroups and whether they differed. The comparison between ECT and Ketamine groups is informative to the research question and analyses conducted.

We have added this to Table 1 as follows:

AUDIT-3 subgroups	*Score 0: 74%*<br>*Score 1: 20%*<br>*Score 2: 4%*<br>*Score 3: 1%*<br>*Score 4: 1%*	*Score 0: 69%*<br>*Score 1: 19%*<br>*Score 2: 6%*<br>*Score 3: 4%*<br>*Score 4: 2%*	*0.142*

We have also added a new table with more detail:

Table 2: *Demographics by AUDIT-3 subgroup*

Ketamine					
AUDIT-3 score	*0 (n=172)*	*1 (n=41)*	*2 (n=8)*	*3 (n=2)*	*4 (n=2)*
Age ± SD	*46.7 ± 16.9*	*32.3 ± 12.9*	*35.0 ± 13.7*	*50 and 24*	*59 and 43*
Gender	*52% female*<br>*42% male*<br>*3% other*<br>*2% unknown*	*63% female*<br>*34% male*<br>*2% other*<br>*0% unknown*	*25% female*<br>*75% male*	*1 female, 1 male*	*1 female, 1 male*
Baseline QIDS score ± SD	*19.6 ± 6.6*	*20.8 ± 6.0*	*17.3 ± 5.4*	*25 and 25*	*29 and 27*
Sample size by time point	*Time 1: n= 140*<br>*Time 2: n= 123*<br>*Time 3: n= 117*<br>*Time 4: n= 96*<br>*Time 5: n= 96*	*Time 1: n= 37*<br>*Time 2: n= 35*<br>*Time 3: n= 30*<br>*Time 4: n= 28*<br>*Time 5: n= 26*	*Time 1: n= 8*<br>*Time 2: n= 8*<br>*Time 3: n= 7*<br>*Time 4: n= 6*<br>*Time 5: n= 4*	*Time 1: n= 2*<br>*Time 2: n= 2*<br>*Time 3: n= 2*<br>*Time 4: n= 2*<br>*Time 5: n= 2*	*Time 1: n= 2*<br>*Time 2: n= 2*<br>*Time 3: n= 2*<br>*Time 4: n= 2*<br>*Time 5: n= 2*
ECT					
AUDIT-3 score	*0 (n=1796)*	*1 (n=477)*	*2 (n=143)*	*3 (n=107)*	*4 (n=54)*
Age ± SD	*48.6 ± 16.7*	*36.2 ± 13.7*	*36.8 ± 13.7*	*35.5 ± 13.5*	*43.2 ± 13.2*

Gender	*57% female*<br>*41% male*<br>*1% other*<br>*1% unknown*	*54% female*<br>*44% male*<br>*1% other*<br>*1% unknown*	*43% female*<br>*55% male*<br>*1% other*<br>*1% unknown*	*51% female*<br>*47% male*<br>*1% other*<br>*1% unknown*	*48% female*<br>*52% male*
*Baseline QIDS score ± **SD***	*21.1 ± 6.7*	*22.5 ± 6.6*	*21.8 ± 6.6*	*23.4 ± 5.8*	*22.5 ± 6.8*
Sample size by time point	*Time 1: n= 1538*<br>*Time 2: n= 1067*<br>*Time 3: n= 799*	*Time 1: n= 423*<br>*Time 2: n= 310*<br>*Time 3: n= 230*	*Time 1: n= 127*<br>*Time 2: n= 96*<br>*Time 3: n= 60*	*Time 1: n= 93*<br>*Time 2: n= 59*<br>*Time 3: n= 48*	*Time 1: n= 50*<br>*Time 2: n= 37*<br>*Time 3: n= 20*

Reviewer #3: The paper aims to investigate the association between episodes of heavy drinking in the past year with antidepressant efficacy of ketamine. This question is quite important and novel (since past studies have found that family history of AUD can predict antidepressant effectiveness of ketamine). Using a retrospective cohort, where they looked at subjects undergoing treatment with ketamine (and those undergoing treatment with ECT) and grouping patients by their baseline AUDIT-3 scores, the authors examined associations with treatment response. They concluded that patients with trMDD with more frequent episodes of heavy drinking in the past year responded better to ketamine than those with lower frequency. This pattern wasn't seen in ECT patients.
The main limitations of this paper are that the methodology and rationale is not sufficiently described for both Ketamine and ECT treatments. This is important and clinical protocols may vary by facility and thus the specific guidelines/ thresholds/ doses/frequency and decision points as it pertains to this paper would be important to disclose.

We appreciate the reviewer's positive comments about the novelty and importance of our manuscript. We have addressed the reviewer's comments about methodology and rationale below:

Some major comments are listed below:
1. Describe the Ketamine clinical protocol in more detail- what was the frequency of the treatments; what factor was used to determine dose escalation; why was 3/4/5 infusions chosen as end point.
If someone did not respond to these treatments were they discontinued?

We have addressed this with the new text below:
For the present analysis, treatment outcomes were considered after three, four, and five infusions. This range was chosen because at least three infusions were conducted in the challenge phase for most participants, and most patients experience benefit with ketamine within 4-5 infusions. Further time points were not included due to risk of selection bias, as patients who remit after 3-5 infusions may be less likely to receive a sixth infusion, while patients who experience no benefit after 3-5 infusions may also be less likely to receive a sixth infusion.

2. Describe the ECT protocol- what end points were chosen and why: the QIDS was administered every 5 treatments per the manuscript.

We have clarified with the new text below:

The QIDS-SR was used as a quantitative marker to compare mood between treatment sessions. It was administered every 5 treatments for monitoring of outcomes as part of routine clinical practice. For the present analysis, outcomes were analyzed at the first time point (5 treatments) to parallel the ketamine analysis, and were also measured at subsequent time points (10, 15, and 20 treatments) to account for delayed response, which often occurs with ECT.

3. What % improvement in QIDS was deemed as response: was this the same for Ketamine and ECT?

We have clarified that no specific cutoff was chosen for response – the primary analysis was a repeated-measures ANOVA with QIDS scores treated as a continuous outcome:

QIDS-SR scores were treated as a continuous variable rather than choosing a specific cutoff for response.

We have also clarified that no specific QIDS cutoff was used for defining response for the purpose of deciding whether to adjust a patient's dose or protocol; rather, the decision was made based on overall clinical assessment (new text underlined):

After the Challenge phase, patients demonstrating substantive responses on objective scales and subjective psychiatric evaluation continued to complete 8 total infusions. Substantive response was defined based on multifactorial clinical assessment, not based on specific scores on the QIDS-SR.

…

In cases of inadequate response, some patients were switched to brief pulse unilateral ECT or bifrontal or bilateral placement depending on urgency, tolerability, and severity of illness. Inadequate response was defined based on multifactorial clinical assessment, not based on specific scores on the QIDS-SR.

4. Substance use disorder was an exclusion for Ketamine but not for ECT- please describe the % of AUD patients and their characteristics?

Detailed diagnostic phenotyping of AUD was not available in the present dataset, which is why AUDIT-3 was used as a proxy. We have described this in the limitations section (new text underlined):

Ketamine is also more novel and more expensive, which may lead to greater nonspecific effects such as expectation bias. It should also be noted that we have distinguished between Episodic Heavy Alcohol Use and Alcohol Use Disorder; full diagnosis of AUD is difficult to assess because patients with comorbid AUD have been either systematically excluded or underrepresented in many prospective studies of ketamine (Murrough et al. 2013; Daly et al. 2018). This trend also applies the present sample, which excluded patients who met formal criteria for an active substance use disorder <u>in the ketamine group and did not systematically assess AUD diagnosis in the ECT group</u>.

5. It appears that overall, the percentage of responders (black line) in non-drinkers was lower in. ketamine compared to ECT driving the difference rather than a greater improvement in "drinkers".

We agree that ECT appeared to be more effective than ketamine for non-drinkers in this sample. However, we did not make direct statistical comparisons between ECT and ketamine in this study because of many potential sources of selection bias. The comparison here is not between ketamine and ECT, but rather between drinkers and non-drinkers in the ketamine group. We have clarified as below (new text underlined):

These results also may shed light into the ongoing quest to best allocate ketamine versus ECT in different contexts. A recent clinical trial showed that ketamine was noninferior to ECT for nonpsychotic trMDD (Anand et al. 2023). While the ECT protocol used in this study may not have been optimal and there was a wide difference in dropout rate (Ekstrand et al. 2024), questions still remain regarding how to determine which treatment is optimal for a specific patient. Given that MDD and AUD are often comorbid(Sullivan et al. 2005; Castillo-Carniglia et al. 2019), a history of episodic drinking in the year before potential treatment may constitute one element considered in the evaluation of patients for treatment with ketamine. <u>Although we did not directly compare ECT to ketamine in the present analysis due to many potential sources of selection bias, patients with no history of heavy drinking appeared to respond better to ECT, while those with some history of heavy drinking appeared to respond better to ketamine</u>.

The reviewer mentions that the differences between the ECT group and the ketamine group may be “driving the difference.” If we understood the reviewer’s comment correctly, it appears to be an error - the primary difference was between drinkers and non-drinkers in the ketamine group, and thus it is impossible that anything about the ECT group could be driving the difference. Hopefully our revisions above will help clarify this, but if we misunderstood the reviewer’s comment, we would be happy to make additional revisions to address it.

There are also some other specific comments:

* Page 6, line 9: Does this mean that patients with episodic heavy alcohol use with and without current AUD were included in the ECT cohort? What was the proportion of these patients? Since, the ketamine cohort only had patients with episodic heavy alcohol use and no current AUD, would it make sense to compare the same with the ECT group?

As stated above, systematic diagnostic phenotyping of AUD was not conducted. We have added this to the limitations as follows:

Ketamine is also more novel and more expensive, which may lead to greater nonspecific effects such as expectation bias. It should also be noted that we have distinguished between Episodic Heavy Alcohol Use and Alcohol Use Disorder; full diagnosis of AUD is difficult to assess because patients with comorbid AUD have been either systematically excluded or underrepresented in many prospective studies of ketamine (Murrough et al. 2013; Daly et al. 2018). This trend also applies the present sample, which excluded patients who met formal criteria for an active substance use disorder in the ketamine group and did not systematically assess AUD diagnosis in the ECT group.

* Table 1:

o Why is race/ethnicity row blank?

We have corrected this:

Race/ethnicity *(multiple selections allowed)*	*89% White<br>1% Native American<br>3% Asian/Pacific Islander<br>2% Black<br>3% Latino<br>2% Other*	*92% White<br>1% Native American<br>6% Asian/Pacific Islander<br>0.5% Black<br>2% Latino<br>2% Other*	*n/a*

o What are the different timepoints. A brief description of each timepoint will make the table easier to understand

We have revised the table to describe the actual number of treatments to be more clear:

Time point	*Baseline (time 0): n=2,588<br>5 treatments: n= 2,350<br>10 treatments: n= 1,851<br>15 treatments: n= 1,357*	*Baseline (time 0): n= 225<br>1 treatment: n= 195<br>2 treatments: n= 197<br>3 treatments: n= 178<br>4 treatments: n= 150<br>5 treatments: n= 152*	*n/a*

o Why was timepoint 8 (or end of treatment) not included?

Further assessments were not included due to high risk of selection bias. Patients who improve are more likely to discontinue treatment early. Although we have already mentioned this in response to the reviewer's request (above) for more details about treatment intervals, we have also restated it below for the reviewer's convenience:

For the present analysis, treatment outcomes were considered after three, four, and five infusions. This range was chosen because at least three infusions were conducted in the challenge phase for most participants, and most patients experience benefit with ketamine within 4-5 infusions. Further time points were not included due to risk of selection bias, as patients who improve after 3-5 infusions may be less likely to receive a sixth infusion.

o Maybe have a similar table with the AUDIT groups? Was there any gender difference in different AUDIT-3 groups.

We have added the requested table:

Table 2: *Demographics by AUDIT-3 subgroup*

Ketamine					
AUDIT-3 score	*0 (n=172)*	*1 (n=41)*	*2 (n=8)*	*3 (n=2)*	*4 (n=2)*
Age ± SD	*46.7 ± 16.9*	*32.3 ± 12.9*	*35.0 ± 13.7*	*50 and 24*	*59 and 43*
Gender	*52% female<br>42% male<br>3% other<br>2% unknown*	*63% female<br>34% male<br>2% other<br>0% unknown*	*25% female<br>75% male*	*1 female, 1 male*	*1 female, 1 male*
Baseline QIDS score ± ***SD***	*19.6 ± 6.6*	*20.8 ± 6.0*	*17.3 ± 5.4*	*25 and 25*	*29 and 27*
Sample size by time point	*Time 1: n= 140<br>Time 2: n= 123<br>Time 3: n= 117<br>Time 4: n= 96<br>Time 5: n= 96*	*Time 1: n= 37<br>Time 2: n= 35<br>Time 3: n= 30<br>Time 4: n= 28<br>Time 5: n= 26*	*Time 1: n= 8<br>Time 2: n= 8<br>Time 3: n= 7<br>Time 4: n= 6<br>Time 5: n= 4*	*Time 1: n= 2<br>Time 2: n= 2<br>Time 3: n= 2<br>Time 4: n= 2<br>Time 5: n= 2*	*Time 1: n= 2<br>Time 2: n= 2<br>Time 3: n= 2<br>Time 4: n= 2<br>Time 5: n= 2*
ECT					
AUDIT-3 score	*0 (n=1796)*	*1 (n=477)*	*2 (n=143)*	*3 (n=107)*	*4 (n=54)*
Age ± SD	*48.6 ± 16.7*	*36.2 ± 13.7*	*36.8 ± 13.7*	*35.5 ± 13.5*	*43.2 ± 13.2*
Gender	*57% female<br>41% male<br>1% other<br>1% unknown*	*54% female<br>44% male<br>1% other<br>1% unknown*	*43% female<br>55% male<br>1% other<br>1% unknown*	*51% female<br>47% male<br>1% other<br>1% unknown*	*48% female<br>52% male*
Baseline QIDS score ± ***SD***	*21.1 ± 6.7*	*22.5 ± 6.6*	*21.8 ± 6.6*	*23.4 ± 5.8*	*22.5 ± 6.8*
Sample size by time point	*Time 1: n= 1538<br>Time 2: n= 1067<br>Time 3: n= 799*	*Time 1: n= 423<br>Time 2: n= 310<br>Time 3: n= 230*	*Time 1: n= 127<br>Time 2: n= 96<br>Time 3: n= 60*	*Time 1: n= 93<br>Time 2: n= 59<br>Time 3: n= 48*	*Time 1: n= 50<br>Time 2: n= 37<br>Time 3: n= 20*

* Page 7, line 7: What was the number of participants in each group with each AUDIT-3 score in the different timepoints. Since, some subjects who didn't show substantive response discontinued after 3-4 treatments, and only 49 patients had AUDIT-3 > 0, this can be an important piece of evidence.

We have added this to Table 2 (please see the response to the previous comment).

* Figure 1:

o What is percent improvement? Is it percent improvement from baseline or from the previous timepoint? Is it the mean percent improvement across all participants (or the median)?

We have clarified this in the figure legend (new text underlined):

Figure 1: Prior episodic heavy alcohol use predicts antidepressant response to ketamine, but not ECT. (a) *With ketamine, patients with a history of past-year episodes of heavy drinking (colored lines) showed greater percent improvement in depression from baseline relative to those without (black line) at time points 3, 4, and 5.*

o The way timepoints are labelled across the x-axes for ketamine and ECT are different (and different from what's mentioned in the table).

We have improved clarity by referring to the actual number of treatments (rather than the corresponding time point) throughout the tables, figures, and main text.

o The way y-axes are drawn are different (the ET groups have percentages mentioned as well)

The figure has been revised to put "percent improvement" as the y-axis label and the same units/scale between panels A/C and between panels B/D.

o There is a rapid improvement in patients with AUDIT-3 = 3 in the ketamine cohort. Does that mean that these patients responded to the initial doses of ketamine (0.5mg/kg) and then continued the trend? What were the doses for the AUDIT-3 = 4 subgroup?

Unfortunately dosing was not recorded in the database analyzed for this study, but we have added sample sizes to the figure to clarify that the reviewer's observation refers to a group that only contained two patients. We have also clarified in the limitations section that some of the individual AUDIT-3 groups had very small sample sizes, so results within those

groups are likely not interpretable; for this reason, the primary analyses were conducted across groups to increase power (see below; new text underlined).

The retrospective nature of this study comes with limitations. A direct comparison between ECT and ketamine was not conducted as we would be unable to control for selection bias. For instance, the patients may differ in terms of treatment resistance, severity, and socioeconomic status amongst other variables. Ketamine is also more novel and more expensive, which may lead to greater nonspecific effects such as expectation bias. It should also be noted that we have distinguished between Episodic Heavy Alcohol Use and Alcohol Use Disorder; full diagnosis of AUD is difficult to assess because patients with comorbid AUD have been either systematically excluded or underrepresented in many prospective studies of ketamine (Murrough et al. 2013; Daly et al. 2018). This trend also applies the present sample, which excluded patients who met formal criteria for an active substance use disorder. As a result, only a small number of patients had very high AUDIT-3 scores; thus, trends within each AUDIT-3 subgroup were not analyzed due to inadequate power.
