# Supplementary material for: History of episodic heavy alcohol use predicts antidepressant effectiveness of ketamine
Source: Psychopharmacology (Berl). 2025 Oct 8;243(7):1693–9. doi: 10.1007/s00213-025-06918-4 (PMC13284259; doi:10.1007/s00213-025-06918-4)
Supplement: Supplementary file 2 [file 213_2025_6918_MOESM2_ESM.pdf]

August 26, 2024
P. Brambilla PhD & J.C. Soares MD, PhD
Psychopharmacology

Dear Editors-in-Chief:

We are writing to submit our manuscript titled "**History of episodic heavy alcohol use predicts antidepressant effectiveness of ketamine**" for consideration for publication in the *Journal of Affective Disorder*s.

In our study, we demonstrated that a history of heavy episodic alcohol use could serve as a predictive marker for antidepressant effectiveness of ketamine treatment. This effect was specific to ketamine relative to ECT. This is consistent with multiple studies showing that ketamine is more effective for depression in patients with a family history of alcohol use disorder, but to our knowledge, it is the first time that this has been extended to a personal history of alcohol use.

Given the *Journal of Affective Disorder'*s commitment to advancing treatment for major depression, we believe our manuscript may interest your readership. The study underscores the importance of individual patient histories in treatment planning and supports a more tailored approach to managing treatment-resistant depression. It also may provide a foundation for further investigation of specific subgroups of patients who are more sensitive to NMDA receptor modulation, a key part of the mechanisms of both ketamine and alcohol.

We appreciate your consideration of our manuscript.

Sincerely,

Aaishah Raquib, OMS III
Western University College of Osteopathic Medicine
200 Mullins Dr, Lebanon, OR 97355
Contact Phone Number: 205-401-7511
Contact Email: aaishah.raquib@westernu.edu
